# A Scoping Review of the Apparent Phenomenon of the Improvement in Hypoparathyroidism in Pregnant and Postpartum Females

**DOI:** 10.7759/cureus.46123

**Published:** 2023-09-28

**Authors:** Maxim John Levy Barnett

**Affiliations:** 1 Internal Medicine, Einstein Medical Center Philadelphia, Philadelphia, USA

**Keywords:** parathyroid hormone-related peptide, calcitriol toxicity, primary hypoparathyroidism, lactation, hypercalcemic crisis

## Abstract

Hypoparathyroidism requires management with both calcium supplementation and active vitamin D to avert a state of hypocalcemia. During late gestation and the postpartum period (specifically lactation), there is an under-recognized, yet intriguing occurrence of apparent ‘pseudohyperparathyroidism’, whereby supplementation dosages may need to either be reduced or discontinued, to prevent hypercalcemia. The explanation for this apparent phenomenon of improved parathyroid status (‘remission’ or ‘resolution’) is incompletely understood; the purpose of this review is to analyze the case reports of this enigma within the medical (and grey) literature, providing an overall pathophysiological explanation and recommendation for the management of such patients. A literature search was conducted through PubMed/Medline, CINAHL, Cochrane Library Database, Scopus, UpToDate, Google Scholar, and the grey literature without a time-restricted period, analyzing all available articles within the literature describing an apparent improvement in parathyroid status in late-gestation and postpartum (lactating) females. Non-hypoparathyroid case reports were also included to further analyze and synthesize an overall likely pathophysiological explanation. Through the literature search, 24 papers were identified covering such a phenomenon in patients with hypoparathyroidism, alongside multiple additional reports of a similar occurrence in patients without underlying hypoparathyroidism. The pathophysiology is believed to occur due to the placental production of parathyroid hormone-related peptide (PTHrP) during gestation, with further production from the lactating mammary glands during the postpartum period. A typical pattern is observed, with increased PTHrP and suppressed PTH throughout both gestation and lactation (present in both normal and hypoparathyroid subjects). The concept of PTHrP-induced hypercalcemia is further demonstrated in patients without hypoparathyroidism, including subjects with placental hypersecretion and mammary gland enlargement. It is evident that patients with hypoparathyroidism may require a dosage reduction during late gestation and lactation, due to the risk for hypercalcemia. In addition to patients with hypoparathyroidism, this pathophysiological phenomenon occurs in unsuspecting patients, demonstrating the need for all clinicians in contact with pregnant females to be aware of this uncommon - yet perilous - occurrence.

## Introduction and background

Initially described in the great Indian rhinoceros by Sir Richard Owen in 1852, the parathyroid glands were an addition to the knowledgebase in the ever-evolving field of endocrinology [1]. Succeeding Sir Owen’s discovery, the existence of parathyroid glands in humans was depicted by Remark (1855) and Sandström (1880) [1]. Throughout the following century, Gley (1891), Moussu (1898), MacCallum and Voegtlin (1909), and Berkeley and Beebe (1909) consecutively identified the effects of the parathyroid gland and its temporal relationship to plasma calcium, noting a demise with the removal of this unique gland [1,2]. The first documentation of successful isolation of the parathyroid hormone (PTH, parathormone) was noted in 1925 by Collip and further refined by Rasmussen and Craig in 1959 [1].

**Figure 1:**
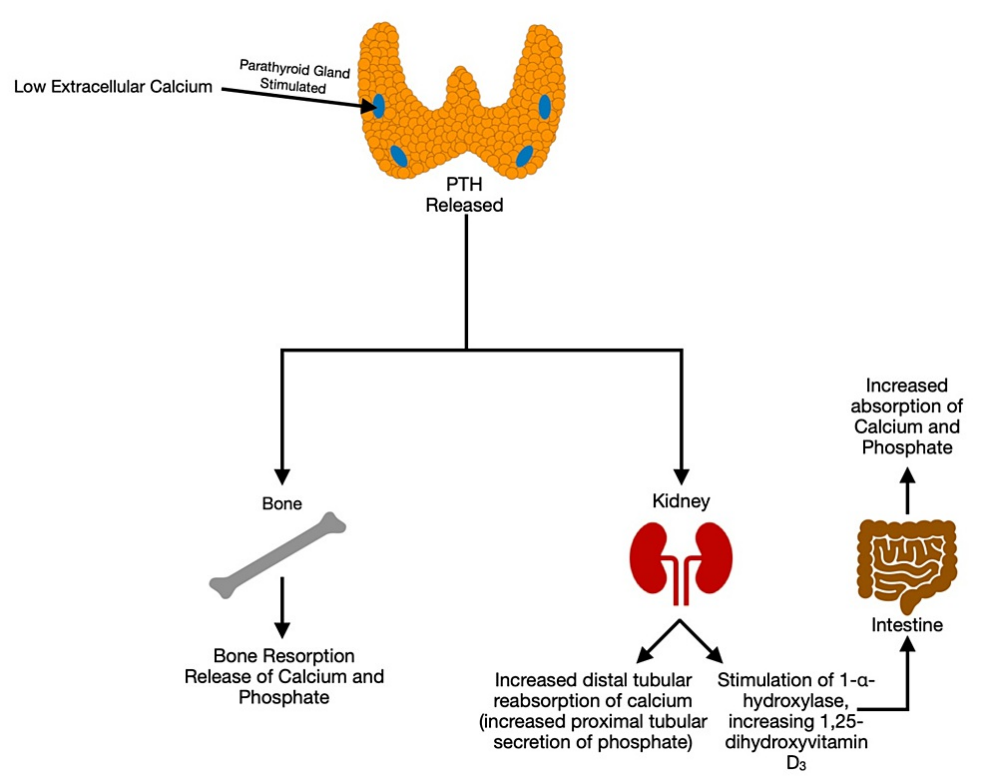
Physiology of Parathyroid Hormone and Hypocalcemia

PTH is an 84-long amino acid polypeptide, released from the chief cells of the four parathyroid glands (two superoposterior and two inferoposterior to the thyroid bed), functioning as a primary regulator of serum calcium homeostasis. In the setting of elevated extracellular calcium (hypercalcemia), the release of PTH is inhibited; conversely, low extracellular calcium (hypocalcemia) leads to the release of PTH. The actions of PTH are multifold and include enhanced bone resorption, increased distal tubular reabsorption of calcium, and enhanced renal production of 1-α-hydroxylase (Figure 1).

Hypoparathyroidism, colloquially referred to as ‘underactive’ parathyroid gland(s), is characterized by an inadequate release of PTH necessary to maintain calcium equilibrium - this is demonstrated by hypocalcemia and either a low or inappropriately normal PTH measurement. Whilst multiple etiologies may be responsible for the diagnosis of hypoparathyroidism, iatrogenic causes (in relation to a prior neck operation) are most common (Table 1) [3].

**Table 1:** Etiologies of Hypoparathyroidism

Etiologies of Hypoparathyroidism
Post-surgical
Autoimmune
Idiopathic
Radiation
Genetic
Infiltrative (Metastases)
Hypomagnesemia
Hypermagnesemia

Hypoparathyroidism has a prevalence of up to 6.6% in the general population, encompassing all age groups, but lacks a specific gender preponderance [4,5]. The principal management of hypoparathyroidism is to prevent the sequelae of untoward hypocalcemia; this is managed through the prescription of lifelong active vitamin D alongside calcium supplementation. With pregnant patients harboring a diagnosis of hypoparathyroidism, supplemental requirements may vary throughout each trimester, requiring an individualized approach to prevent feto-maternal complications [6] (Table 2).

**Table 2:** Effects of Hypoparathyroidism Upon Gestation

Effects of Hypoparathyroidism Upon Gestation
Preterm delivery
Miscarriage
Fetal skeletal demineralisation
In-utero fractures
Fetal respiratory distress
Fetal death
Neonatal secondary hyperparathyroidism

During the postpartum period, an apparent phenomenon is noted, whereby lactating females (with hypoparathyroidism) may demonstrate an apparent state of calcitriol toxicity (hypercalcemia) requiring a temporary reduction (or discontinuation) of their medications. This is characterized by subversion into an apparent temporary remission (or improvement) of hypoparathyroidism and has also been documented during late gestation. As this occurrence is infrequently recorded, the focus of this article is to reinforce this pivotal concept, as well as to provide an overall recommendation for the management of such patients.

Outside the field of endocrinology, the quandary for calcitriol toxicity (temporary hypoparathyroidism resolution) during lactation is unlikely to be common knowledge. Parous women with a diagnosis of hypoparathyroidism are likely to attend appointments with specialties other than endocrinology - examples include obstetrics, midwifery, and general practitioners. This demonstrates multiple occasions for potential proactive maternal care. Due to the limited understanding of this phenomenon, coupled with the potential for preventable maternal harm, the purpose of this review is to broaden the awareness of the potential for hypercalcemia in postpartum, lactating females, with an established diagnosis of hypoparathyroidism.

The primary objective of this review is, therefore, to

1. Provide a scoping review of the medical literature pertaining to the phenomenon of an ‘improvement’ or ‘remission’ of hypoparathyroidism during late gestation and lactation, with the intent of enhancing awareness of this potential (harmful) outcome across further medical specialties (and afflicted patients).

The secondary objectives of this review are to

1. Provide an overview of the proposed pathophysiological hypotheses pertaining to the apparent improvement in hypoparathyroidism (and potential for calcitriol toxicity).

2. Review the recommendations for the management of hypoparathyroidism in late gestation and the postpartum period.

Whilst the temporary resolution of hypoparathyroidism is described in the medical literature, this is mainly limited to case reports and case series. Due to the paucity of data, this review is justified, with the intent to provide awareness to the greater medical community of the potential for calcitriol toxicity (requiring dosage revision) during both late gestation and postpartum (lactation). Ensuring that a broader group of healthcare professionals (and motivated patients) are aware of this potential outcome will safeguard proactive patient care and result in a reduction in preventable feto-maternal harm.

## Review

Materials and methods

This review is delivered as a scoping review, encompassing the broad medical literature, with a subset focus on relevant case reports/series from the ‘grey’ literature, which further substantiates the apparent (under-studied) phenomenon of hypoparathyroidism improvement during late gestation and in postpartum, lactating females. Providing a scoping review allows for a multi-faceted approach in the deciphering of this apparent endocrine anomaly, covering multiple case documentation, reviewing (and synthesizing) the proposed pathophysiology, providing rationales for the recommended management of this unique subset of patients, and highlighting areas of uncertainty.

Search Strategy

Whilst, in research, the highest-regarded bodies of evidence (to inform clinical practice) are randomized controlled trials, meta-analyses, and systematic reviews, infrequent (albeit important) medical occurrences (which have the potential to change medical practice) are often limited to less-rigorous bodies of evidence. For this reason, this review encompasses all forms of articles published (such as case reports, case series, expert opinion, cohort studies, randomized controlled trials, meta-analyses, and systematic reviews). In addition to the variable article types available, to prevent further exclusion of informative data, the electronic search conducted did not have a time-restricted period. The search strategy entailed Cochrane Library Database, Scopus, PubMed/MEDLINE, the Cumulative Index to Nursing and Allied Health Literature (CINAHL), UpToDate, and Google Scholar (in addition to the grey literature). The following keywords were used in the retrieval of available literature pertaining to the principal topic of this professional project: “hypoparathyroid” AND “lactation” OR “hypoparathyroidism” AND “lactation” OR “hypoparathyroidism” AND “breastfeed” OR “hypoparathyroidism” AND “remission” AND “lactation” OR “hypoparathyroid” AND “remission” AND “postpartum” OR “hypoparathyroidism” AND “resolution” AND “lactation” OR “calcitriol treatment decreased” AND “hypoparathyroid” AND “lactation”.

Publications in languages other than English were included (four case reports - one in Japanese, one in German, and two in French). The search included reports of this occurrence without hypoparathyroidism to further support the pathophysiological hypotheses. Male subjects were excluded from the search strategy; animal studies were included in the search strategy, to be incorporated under the pathophysiological section of this professional project (however, animal case series/reports are not analyzed). The focus of inclusion is postpartum (lactating) females. However, to further reiterate the pathophysiological hypotheses, non-lactating, postpartum females (and those during gestation) were also included. Further, articles were included from the reference list of an initial article identified (when relevant to the aforementioned inclusion criteria). Both full-text articles and abstracts (whereby the case description was apparent and data could be deciphered) were included (Table 3 for the inclusion and exclusion criteria).

**Table 3:** Inclusion and Exclusion Criteria

Inclusion Criteria	Exclusion Criteria
Female subjects with (or without) hypoparathyroidism	Male subjects with (or without) hypoparathyroidism
Human subjects	Non-human subjects
Articles whereby full text is available (or abstract provides decipherable data)	Articles whereby full text is not available (or abstract does not provide decipherable data)
Gestation, postpartum (+/- Lactation), or neither pregnant nor postpartum	
Articles cited in the reference list of the initial article	
Publication in any language	

*Data Extraction and Analysis*

**Figure 2:**
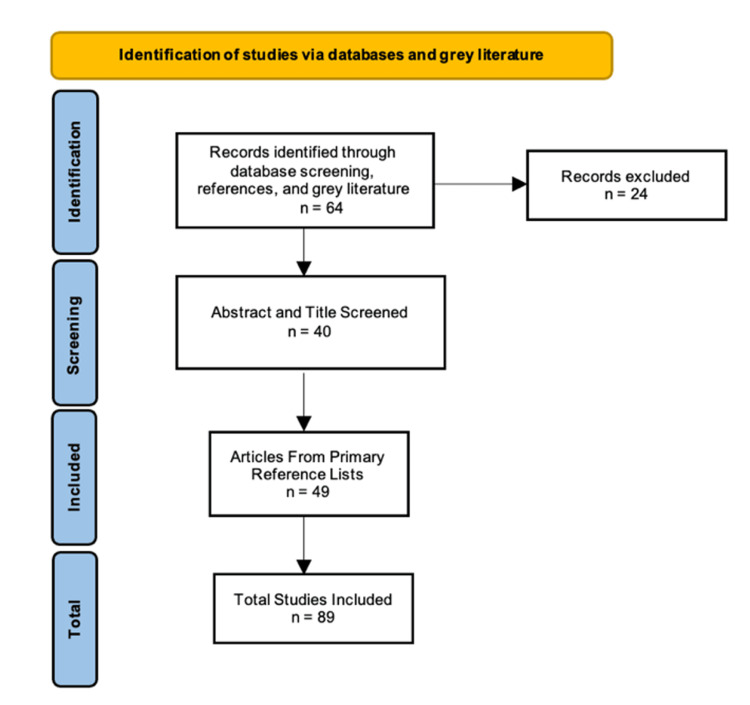
PRISMA Flowchart

The aforementioned search strategy yielded n = 64 citations from the databases; of these, n = 40 citations describing hypercalcemia in both hypoparathyroid and pseudohypoparathyroid subjects were identified after screening the title and abstracts (and applying the inclusion/exclusion criteria). A further n = 49 citations were identified from primary reference lists and used to further the pathophysiological explanation of this professional project (Figure 2).

Quality of Evidence

As the purpose of this scoping review is to synthesize the cases relating to the apparent improvement in hypoparathyroidism and to analyze areas of uncertainty in current knowledge, a quality appraisal was not performed for this review.

Risk of Bias Assessment

Scoping reviews are prone to bias, especially when considering a rare documented medical occurrence whereby only patients experiencing the phenomenon are likely to be depicted in the literature (an uneventful postpartum period is unlikely to be reported in the literature). There is no data to suggest the percentage of pregnant and lactating postpartum females with hypoparathyroidism who do not experience the enigma of temporary resolution or remission. As this is a scoping review, a risk of bias assessment was not performed.

Results

Wright et al. [7] posed an interesting constellation of three pregnancies (two patients) with hypoparathyroidism, developing postpartum hypercalcemia. In the first patient (first pregnancy), serum calcium remained normal during pregnancy, with adherence to 100,000 IU of calciferol and 57 mEq of daily calcium. These medications were continued for the first three days postpartum, followed by 50,000 IU of calciferol for a further three days. The patient did not breastfeed (prevented with hexoestrol). On postpartum day five, the patient presented with symptomatic hypercalcemia (7.6 mEq/L), requiring immediate discontinuation of all treatment, with serum calcium returning to normal by postpartum day 17. At this time, 76 mEq of elemental calcium was continued for three months, until 15 weeks postpartum, at which time hypocalcemia was demonstrated (3.8 mEq/L) and managed by reintroducing 100,000 IU of calciferol daily.

Two years later, the same patient delivered her second child; again, during her pregnancy, serum calcium was unremarkable, and she was adherent to 100,000 IU of calciferol and 76 mEq of calcium daily. The authors record that, for the second time, she did not breastfeed and note that, within 24 hours postpartum, calcium rose to 6.0 mEq/L, at which point all medications were discontinued. Serum calcium subsequently decreased to 5.1 mEq/L over the following six days postpartum. Calciferol (50,000 IU) and 38-57 mEq/L of calcium were restarted; however, symptomatic hypercalcemia reappeared (6.0 mEq/L), requiring discontinuation on postpartum day 20. Roughly one week following the discontinuation of her medications, the serum calcium decreased to 4.8 mEq/L, at which point half her usual dosage was reintroduced for the succeeding 12 days.

A second patient was described by Wright et al. [7], who was managed with calciferol (150,000 IU daily) throughout pregnancy, with no apparent hypercalcemia. After delivery, lactation was suppressed with hexoestrol, and it is noted that, on postpartum day one, calcium rose to 5.7 mEq/L and continued to increase (6.8 mEq/L on day six postpartum). The patient was asymptomatic and, against medical advice, continued her calciferol throughout the postpartum period.

In contrast to Wright et al. [7], Sadeghi-Nejad et al. [8] described a patient with hypocalcemia during pregnancy, requiring increasing dosages of calcitriol (1.5-3.0 μg daily) to maintain serum calcium at 8 mg/dL. Similar to Wright et al. [7], the patient declined to breastfeed; her calcitriol dosage was reduced to pre-pregnancy requirements (1.5 μg per day), with serum calcium remaining at the upper end of normal (10 mg/dL). These findings suggest an improvement in calcitriol sensitivity in the postpartum period, replicating the findings of Wright et al. [7], occurring even in the absence of lactation.

Markestad et al. [9] presented a 23-year-old female with hypoparathyroidism, managed with two micrograms of 1-α-hydroxyvitamin D (alfacalcidol) (requiring a dosage increase in early pregnancy and temporary calcium supplementation), who commence breastfeeding following delivery. At 16 days postpartum, she presents with symptomatic hypercalcemia (4.10 mmol/L) (16.43 mg/dL), and her two micrograms of alfacaldiol are discontinued. The patient’s serum calcium normalized following the cessation of breastfeeding, the introduction of intravenous fluids, diuretics, and the replacement of alfacalcidol with 30-90,000 IU of vitamin D2 per day.

Rude et al. [10] presented a case report similar to Markestad et al. [9], describing a lactating 26-year-old female with hypoparathyroidism. The patient was managed with 0.5 μg of calcitriol per day, and on the second postpartum day, her serum calcium increased to 10.0 mg/dL, requiring discontinuation of calcitriol. The patient breastfed for four months, whereby no calcitriol was required during this time (maintaining serum calcium around 9.5 mg/dL). The authors note that, one week after cessation of breastfeeding, she presented with symptomatic hypocalcemia (7.5 mg/dL), requiring the reintroduction of calcitriol (up to 2.5 μg per day).

Cundy et al. [11] describe a 26-year-old female with hypoparathyroidism (managed with 0.5 μg of calcitriol thrice daily) who delivered at 41 weeks gestation. The patient commences lactation and is noted to present on day nine postpartum with hypercalcemia (3.32 mmol/L) (13.31 mg/dL), requiring discontinuation of calcitriol, for a total of 40 days (followed by the small reintroduction of calcitriol). The authors note that, very soon following weaning, pre-pregnancy dosages of calcitriol were required.

Caplan et al. [12] presented a similar finding, whereby continuation of calcitriol during lactation in a hypoparathyroid female (age not revealed) led to hypercalcemia on postpartum day 11 (15.4 mg/dL); in the patient’s second pregnancy (reported by Caplan et al. [13]), a pre-emptive reduction of calcitriol (0.75-0.25 μg per day) during lactation maintained low-normal serum calcium (noting requirements of 0.75 μg per day following weaning). Caplan et al. [12] further assessed two other women (four pregnancies), noting hypercalcemia did not occur in patients who had a pre-emptive reduction in active vitamin D (calcitriol or dihydrotachysterol) after delivery or who did not breastfeed. Richa et al. [5] replicated this ‘proactive reduction’ in 45-year-old women with hypoparathyroidism at two weeks postpartum, maintaining stable serum calcium around 8.4 mg/dL with a reduction in calcitriol from 1.0 to 0.5 μg per day (with frequent caltrate adjustments during lactation).

Höper et al. [14] presented a 23-year-old female with partial DiGeorge syndrome, noting hypercalcemia (2.7 mmol/L) (10.82 mg/dL) at week 37 of gestation, requiring a reduction in the dosage of calcitriol (dosage not stated). The authors meekly note that the serum calcium continued to rise postpartum, recorded on the 12th day, but make no mention of whether the patient was lactating. This latter drawback, however, is overcome by a presentation [15], with a 28-year-old female with DiGeorge syndrome. The authors note the patient was managed with 0.25 μg of calcitriol daily and 750 mg of calcium carbonate thrice daily and presented at two weeks postpartum (lactating) with hypercalcemia (12.7 mg/dL; during delivery, her calcium was normal at 8.6 mg/dL). Initial management included alternate-day dosing of calcitriol. However, six weeks after this adjustment, she remained hypercalcemic (13.1 mg/dL), requiring discontinuation of calcium carbonate and extending calcitriol to once every three to five days. The authors note that the patient successfully lactated for a further 12 months, with her calcitriol requirements gradually reaching pre-pregnancy requirements upon weaning.

Cathébras et al. [16] further observed this phenomenon in two women with hypoparathyroidism during lactation; the first patient described required between 1.0 and 1.5 mg of calcitriol, alongside 1 g of calcium per day and was noted at two months post-delivery to demonstrate a serum calcium of 3.42 mmol/L (13.71 mg/dL), at which point calcitriol was withdrawn and weaning commenced, allowing serum calcium to normalize (eventually maintained on 0.5 mg of calcitriol per day). The second patient described by Cathébras et al. [16] was managed with 3 mg of alfacalcidol per day and 2 g of calcium, noting that, five days postpartum (whilst breastfeeding), serum calcium was detected at 3.45 mmol/L (13.83 mg/dL); the hypercalcemia rapidly normalized after two days following discontinuation of alfacalcidol (eventually managed with 2 μg per day).

Shomali et al. [17] presented a similar case report during lactation in a 35-year-old female with hypoparathyroidism (managed with 1 mg of dihydrotachysterol (adjusted between 1.4 and 1.6 during the third trimester) and 3 g of calcium carbonate (increased to 4 g during the third trimester)). At delivery, the patient’s calcium was measured to be 8.6 mg/dL, for which the aforementioned medications and respective dosages were continued for another two weeks until postpartum day 18, when she demonstrated severe hypercalcemia (19.2 mg/dL) requiring immediate treatment. Upon discharge, the patient was prescribed 0.5 μg of calcitriol and 2 g of calcium carbonate daily. However, three days later, serum calcium increased from 10.3 mg/dL to 11.4 mg/dL, at which point both supplements were discontinued. The authors note that the patient did not require further supplementation until postpartum day 87, whereby hypocalcemia was present (6.4 mg/dL), which was managed with 0.5 μg of calcitriol and 2 g of calcium carbonate daily.

Anai et al. [18] presented a 35-year-old female with hypoparathyroidism (managed with 1 μg of alfacalcidol daily), who breastfed for six months postpartum (after her first pregnancy), noting a pattern of increasing calcium during lactation (2.44 +/- 0.08 mEq/L), followed by an abrupt decrease during weaning (2.35 +/- 0.08 mEq/L). The authors demonstrated a significantly greater mean ionized calcium duration lactation compared to pregnancy (p < 0.001) and post-weaning (p < 0.05). Three years later, the patient becomes pregnant again, noting a similar presentation of increasing calcium during lactation (2.47 +/- 0.19 mEq/L), decreasing post-weaning (2.18 +/- 0.12 mEq/L), again with a significant pattern of greater mean ionized calcium during lactation compared to pregnancy (p < 0.05) and post-weaning (p < 0.05). Of note, due to the recognized phenomenon occurring during her first postpartum period, her alfacalcidol was pre-emptively discontinued during lactation.

Mather et al. [19] presented a 28-year-old female with hypoparathyroidism who presents with hypercalcemia during lactation requiring discontinuation of supplements during weeks two and six postpartum (dosages not described). During the patient’s second pregnancy, lactation was immediately commenced following delivery and continued for 72 weeks. During the first two weeks, elemental calcium (2 g) was discontinued, followed by calcitriol dosage reductions (0.75 μg per day) until it was discontinued by week seven postpartum; the authors note no supplements were required for 72 weeks postpartum.

Yasumatsu et al. [20] presented two women with hypoparathyroidism demonstrating hypercalcemia during lactation, requiring dosage alterations. The first patient described was managed with 6 g of calcium per day and 2 μg of alfacalcidol with unremarkable serum calcium during pregnancy. The patient underwent a caesarean section and, seven days later, presented with hypercalcemia (13.3 mg/dL), whereby calcium supplementation was discontinued and active vitamin D was reduced to 1 μg, per day (however, hypercalcemia was persistent requiring total discontinuation of alfacalcidol). The second patient described by the authors was managed with the same dosages above throughout pregnancy with stable serum calcium, with the mother undergoing normal labor. Around two months postpartum, however, serum calcium was demonstrated to be elevated at 12.2 mg/dL, for which calcium supplementations were discontinued, eventually requiring cessation of alfacalcidol. The authors note both patients were lactating during the postpartum periods.

Sweeney et al. [21] presented a 34-year-old female with variable calcitriol and elemental calcium supplementations throughout pregnancy commencing lactation on postpartum day two (with 1,500 μg of calcium carbonate twice daily and 0.25 μg of calcitriol daily) whereby her serum calcium was stable between 8 and 9 mg/dL. By postpartum day 22, however, calcium was noted to be 10 mg/dL, at which point calcitriol was discontinued, and calcium carbonate was reduced to 600 mg twice daily.

Krysiak et al. [22] presented a 32-year-old female with hypoparathyroidism during her second pregnancy at week 23, who was admitted with the concern of preterm delivery and administered magnesium sulfate; this subsequently led to hypocalcemic tetany (1.40 mmol/L) (5.69 mg/dL), requiring intravenous calcium gluconate and a recommended regimen of calcium carbonate (3 g) and calcitriol (0.75 μg). The authors recommended against breastfeeding; however, the patient did not adhere to these recommendations; post-delivery at 38 weeks, the patient commenced lactation and required a dosage reduction of calcitriol (0.5 μg) to maintain normocalcemia.

Segal et al. [23] depicted a 27-year-old female with hypoparathyroidism who develops lactational osteoporosis; the patient was managed with 2 μg of alfacalcidol and 2 g of elemental calcium daily (and is noted to have delivered twins). The patient breastfed for three months, until she presented with back pain and was noted to have hypercalcemia (11.5 mg/dL). The patient was advised to discontinue lactating, reduce alfacalcidol to 0.5 and 1.0 μg (alternate day dosing), and introduce 800 IU of cholecalciferol (elemental calcium dosage unchanged) for which calcium decreased to 8.8 mg/dL.

Al-Nozha et al. [24] reported two postpartum incidences of hypercalcemia in a 37-year-old female with hypoparathyroidism. During the first pregnancy, the patient was noted to be hypercalcemic (2.67-2.74 mmol/L) (10.7-10.98 mg/dL) a few days prior to delivery, continuing for two weeks postpartum (requiring adjustments of calcitriol and calcium supplementation). Despite continued breastfeeding, once menses returned, she developed symptomatic hypocalcemia (requiring 2.4 g of calcium and 0.25 μg of calcitriol). She continued breastfeeding for a further nine months until she noted she was pregnant (nine weeks’ gestation); following cessation of breastfeeding, she presented with recurrent symptomatic hypocalcemia (1.85 mmol/L) (7.41 mg/dL), requiring calcium supplementation ranging between 4 and 8 g (and 0.5-1.0 μg of calcitriol). The authors note a gradual reduction in calcium requirements, receiving 4 g by the end of gestation, alongside 0.5 μg of calcitriol. Al-Nozha et al. [24] demonstrated that, during lactation, she remained stable on the reduced dosages of calcium and calcitriol. The authors describe this concept of hypocalcemia following weaning as comparable to ‘hungry bone syndrome’.

Hatswell et al. [6] analyzed 10 pregnancies encompassing six women with hypoparathyroidism, demonstrating differing results during both gestation and the postpartum period. The authors described postpartum hypercalcemia in only two patients, for which both events occurred as their dosages were not pre-emptively reduced after delivery; the remaining eight pregnancies documented included an appropriate reduction in supplementations after delivery, likely explaining why hypercalcemia was not present. The first patient described is a 38-year-old female who presents on postpartum day 21 with a serum calcium of 3.01 mmol/L (12.06 mg/dL), at which point calcitriol is reduced from 1.5 to 1.0 μg daily. The second patient described is noted to have had a normal pregnancy in the past. However, during her second pregnancy, she presented on postpartum day nine with a serum calcium of 4.16 mmol/L (16.67 mg/dL), for which calcium carbonate (1,200 μg) and calcitriol (0.5 μg) were ceased for one week, after which calcium normalized.

Shah et al. [25] demonstrated recurrent hypocalcemia and labile requirements of calcium, calcitriol, and vitamin D3 during the pregnancy of a 38-year-old female with hypoparathyroidism. The authors demonstrate, however, that during lactation, she developed ‘high-normal’ calcemia, requiring a reduction of 400 μg of elemental calcium and a reduction of 200 IU of vitamin D3.

Dixon et al. [26] posed a similar finding of increased requirements during gestation in a female with congenital hypoparathyroidism (homozygous c.68C>A null parathyroid hormone gene mutation) (age not described). During the patient’s first pregnancy, her prescriptions (1.5 μg of calcitriol and 2,400 μg of elemental calcium daily) were switched to 1.5 μg of calcitriol (with discontinuation of calcium supplementation) at 24 weeks of gestation, which was again switched to 2.0 μg of alfacalcidol at 30 weeks. Four weeks later, this was reduced to 1.5 μg of alfacalcidol daily until delivery. The authors note that the patient did not subsequently require calcium nor vitamin D supplementation during breastfeeding until weaning commenced at around six months (at which time symptomatic hypocalcemia emerged).

Discussion

A pattern is noted in the above case series, whereby calcium requirements are rather labile during pregnancy, but tend to decrease postpartum. Marcucci et al. [27] included 28 patients in a retrospective study with either a diagnosis of hypoparathyroidism (n = 25) or pseudohypoparathyroidism (n = 3), demonstrating the overall trends of calcium and vitamin D supplementation requirements during pregnancy and postpartum. Whilst the authors note two patients developed hypercalcemia during lactation (serum calcium 11-13 mg/dL), there is no further discussion of the management course for these patients. When analyzing from the third trimester to six months postpartum, the authors demonstrated a mean decrease in the requirements of calcium supplementation (445.6 μg per day (-24.7%)) and calcitriol (0.1 μg per day (-11.1%)); when compared to the breastfeeding period (average duration of 6.6 +/- 3.8 months), this was measured at 265 mg and 0.1 μg per day, respectively. With the first control post-lactation (in comparison to the third trimester), the mean requirements of calcium and calcitriol were reduced by 310.4 mg (-17%, p = 0.02) and 0.1 μg per day (-12%), respectively; eventually, the dosages returned to pre-pregnancy requirements (time period not described). The authors propose an interesting suggestion, noting perhaps that part of the reason for increased serum calcium during lactation could be due to enhanced maternal awareness of the requirement for an increase in dietary calcium. Whilst medication dosages may require reduction, Marcucci et al. [27] demonstrated an increase in dietary calcium during breastfeeding (820 +/- 268 mg per day) compared to pregnancy (738.9 +/- 211.8 μg per day).

In a case series performed by Hartogsohn et al. [28], 17 pregnancies amongst 12 patients (across Canada and Denmark) with a diagnosis of hypoparathyroidism (10 of whom continue with lactation following delivery) were analyzed. The authors demonstrated that 36% of subjects (n = 5) developed hypercalcemia either by the end of pregnancy or the start of lactation, with a median increase of ionized plasma calcium from 1.20 mmol/L (4.81 mg/dL) (third trimester) to 1.32 mmol/L (5.29 mg/dL) in the postpartum period (p < 0.03). Hartogsohn et al. [28] noted a significant dosage reduction in alfacalcidol during pregnancy and lactation (p < 0.04) and a significant reduction in requirements when comparing the third trimester to lactation (alongside an increase in serum calcium) (p = 0.01). Furthermore, the requirements for vitamin D significantly increase following weaning (p = 0.04), without a significant difference between pre-pregnancy and post-lactation dosages (p = 0.05). Moreover, there was no significant difference between dosages throughout each trimester (p = 0.91). In a similar manner, the authors note a significant change in the dosage of calcium supplementation (p = 0.03), which required a reduction from the third trimester to lactation (p < 0.03), with a significant increase with weaning (p < 0.02). As noted with vitamin D, there was no significant difference in the dosages of calcium supplementation pre-pregnancy and post-lactation (p = 0.60) nor between each trimester of pregnancy (p = 0.43).

Wang et al. [29] presented similar data to Hartogsohn et al. [28] with a retrospective study in China encompassing 25 pregnancies from 19 patients with hypoparathyroidism. During pregnancy, the authors demonstrated that 26.1% of patients experienced an improvement in serum calcium, 17.4% demonstrated a decline, and 13% remained stable. During lactation, the authors noted a significant increase in serum calcium across 41.7% of patients compared to pregnancy (2.57 +/- 0.34 mmol/L (10.3 +/- 1.36 mg/dL) versus 1.99 +/- 0.11 mmol/L (7.98 +/- 0.44 mg/dL), p < 0.001), noting a further 25% demonstrated ‘high-normal’ serum calcium. Similarly, during lactation, Wang et al. [29] noted significantly greater 24-hour urine calcium in 83.3% of lactating women compared to pregnancy (12.28 +/- 5.41 mmol/L (49.22 +/- 21.68 mg/dL) versus 8.63 +/- 3.22 mmol/L (34.59 +/- 12.91 mg/dL), p = 0.013). The authors demonstrated a reduction in both elemental calcium (1331 +/- 219 mg per day versus 1006 +/- 286 mg per day) and active vitamin D (0.80 +/- 0.19 μg per day versus 0.25 +/- 0.18 μg per day) in all these women during lactation.

Proposed Pathophysiology

The apparent improvement in hypoparathyroidism during late gestation and breastfeeding is an incompletely understood, yet potentially harmful occurrence (risking calcitriol toxicity and hypercalcemia) to the untrained clinician. As noted by Winter et al. [30], this apparent hypercalcemia during either gestation or the postpartum period is colloquially termed ‘pseudohyperparathyroidism’ and is understood to be a result of parathyroid hormone-related peptide (PTHrP), a hormone responsible for humoral hypercalcemia of malignancy, first isolated and purified in 1987 [31]. During normal gestation, there is a steady increase in serum PTHrP (initially from the placenta, followed by the mammary tissue(s)), further increasing during lactation (reaching a maximum at six weeks postpartum and diminishing post-weaning) [32]. Conversely, during gestation, there is a decrease in serum PTH (as well as during lactation), which rises post-weaning [32].

**Figure 3:**
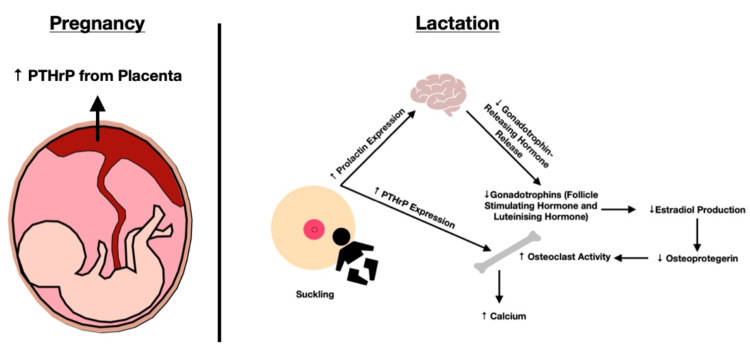
Proposed Pathophysiology

During pregnancy, PTHrP is predominantly released by the placenta (with smaller contributions from the amnion, decidua, fetal parathyroid glands, and umbilical cord). However, there may be concurrent release from enlarged mammary glands during this time period, with a ‘transition’ to release by the mammary glands during the postpartum (lactating) period [33]; these are colloquially described by Lebrun et al. [34] as ‘accessory’ parathyroid glands during gestation and lactation. During lactation, prolactin is released from the anterior pituitary gland; prolactin is known to act upon the gonadotrophin-releasing hormone (GnRH) pulse center in the hypothalamus to suppress the release of gonadotrophins (luteinizing hormone and follicle-stimulating hormone). This furthermore leads to a depletion of estradiol (E2); the presence of low E2 upregulates receptor activator of the nuclear factor kappa beta-ligand (RANKL), which downregulates osteoprotegerin, stimulating osteoclasts and bone resorption (promoting hypercalcemia). As discussed below, hyperprolactinemia is believed to be a stimulus for the release of PTHrP, further contributing to bone resorption and hypercalcemia (Figure 3).

PTHrP

PTHrP is a hormone responsible for humoral hypercalcemia of malignancy and paraneoplastic syndromes (notably squamous cell carcinoma of the lung). It is important to note that, during lactation, PTHrP is released into the breast milk at 1,000-100,000 times the serum concentration that would be present in humoral hypercalcemia of malignancy [19]. During gestation, the function of PTHrP appears to be the placental transfer of calcium to the developing fetus [35]; conversely, during the postpartum period, the purpose of PTHrP appears to be the mobilization of calcium from the maternal skeleton into the breast milk for the neonate. Gallacher et al. [36] suggested that PTHrP may function to regulate mammary blood flow through vasodilatation.

As noted by Kalkwarf et al. [37], a lactating mother will lose around 210 mg of calcium in her breast milk each day. Winter et al. [38] and Wang et al. [29] noted that, in rodents, both the increased intestinal absorption of calcium and skeletal resorption are the predominant mechanisms for calcium acclamation into the breast milk; in humans, however, it appears that only PTHrP (skeletal resorption) is responsible. Both Kovacs [39] and Ali et al. [32] suggested that increased 1,25-dihydroxyvitamin D3 leads to a near-doubling of intestinal calcium absorption during pregnancy; this rate returns to normal during lactation, appearing to correlate with the decline in calcitriol during lactation. Kovacs [39] posed that there is no difference in the fractional calcium absorption between lactating women and controls; as E2 is suppressed during lactation, this allows enhanced bone resorption (in a manner similar to that noted in osteoporotic, post-menopausal females). It is apparent, however, that Kalkwarf et al. [40] demonstrated a 20% increase in absorption of calcium post-weaning (when menses returns and E2 increases); as E2 is elevated during pregnancy, this may explain why there is a presumed doubling of calcium absorption.

Grill et al. [41] demonstrated a consistent rise in serum PTHrP during pregnancy and lactation. The authors analyze n = 18 pregnant patients, n = 19 lactating women, and n = 16 non-lactating women, demonstrating PTHrP being detectable in 63% (12 out of 19) lactating mothers, but none (0 out of 16) of the bottle-feeding mothers, and only one pregnant mother. Conversely, PTH was lower in the lactating mothers (2.3 +/- 1.0 pmol/L) (21.69 +/- 9.43 pg/mL) compared to non-lactating mothers (3.5 +/- 1.2 pmol/L) (33.01 +/- 11.32 pg/mL) (p < 0.01). It is important to note, however, that the sensitivity of the PTHrP assay used by Grill et al. [41] was 2.0 pmol/L; when compared to Gallacher et al. [36] who used a sensitivity of 0.7 pmol/L (6.60 pg/mL), PTHrP is present throughout pregnancy and increases, as demonstrated from the first-trimester measurement (0.8 +/- 0.2 pmol/L) (7.54 +/- 1.89 pg/mL) to six weeks postpartum (2.7 +/- 0.2 pmol/L) (25.46 +/- 1.89 pg/mL) (p < 0.0001).

Similar findings emerged from the study conducted by Kalkwarf et al. [37], noting serum PTH to be 30% lower in lactating women (n = 97) at baseline, compared to non-lactating women (n = 99) (p < 0.001); it should be noted, however, that neither Grill et al. [41] nor Kalkwarf et al. [37] assessed patients with underlying hypoparathyroidism. Kalkwarf et al. [37] introduced a second arm to their study, known as the ‘weaning cohort’, whereby lactating women at six months (n = 95) continued to have lower serum PTH compared to non-lactating women (n = 92) (p < 0.01).

In a longitudinal study performed by Ardawi et al. [42], levels of PTHrP were assessed across three cohorts (without hypoparathyroidism): all trimesters of pregnancy (n = 40), six weeks postpartum (n = 18), and non-pregnant women (n = 280). In 33.9% (n = 95) of the non-pregnant women, PTHrP was undetectable; moreover, the authors describe the levels as ‘very low’ in the remaining patients (0.45 +/- 0.28 pmol/L) (4.24 +/- 2.64 pg/mL) compared to the pregnant and postpartum cohorts. Ardawi et al. [42] demonstrated PTHrP increased from 0.81 +/- 0.12 pmol/L (7.64 +/- 1.13 pg/mL) in the first trimester to 2.01 +/- 0.22 pmol/L (18.95 +/- 2.08 pg/mL) at term. Moreover, PTHrP increased to 2.63 +/- 0.15 pmol/L (24.80 +/- 1.41 pg/mL) at six weeks postpartum (p < 0.001).

In a study of non-hypoparathyroid, lactating women by Dobnig et al. [43], at two-to-three days postpartum (lactation), subjects (n = 58) demonstrated a mean PTHrP nearly double that of the controls (n = 35) (2.64 +/- 0.19 pmol/L (24.90 +/- 1.80 pg/mL) compared to 1.34 +/- 0.14 pmol/L (12.64 +/- 1.32 pg/mL); p < 0.0001). The authors furthermore demonstrate that PTHrP continued to rise throughout lactation (three to six months), albeit levels began to decline, and at six months, PTHrP was significantly lower. Dobnig et al. [43] suggested that around 80% of the controls (and all lactating women) had elevated PTHrP levels. Mather et al. [19] suggested that up to 95% of lactating (and non-lactating, postpartum) women should have an elevated PTHrP if extremely sensitive assays were to be consistently used, likely explaining the discordant findings between Ardawi et al. [42], Grill et al. [41], and Gallacher et al. [36].

While Grill et al. [41] noted elevated PTHrP in 63% (12/19) of nursing mothers, Lippuner et al. [44] demonstrated elevated PTHrP in 100% of nursing mothers (12/12). Lippuner et al. [44] followed 12 nursing women (without hypoparathyroidism) for seven weeks (and 16 weeks post-weaning). During lactation, PTHrP was above the upper limit of normal in all nursing mothers, significantly higher during lactation than weaning (2.8 +/- 0.35 pmol/L (26.40 +/- 3.30 pg/mL) versus 0.52 +/- 0.04 pmol/L (4.90 +/- 0.38 pg/mL), p = 0.002), with a reciprocal decrease in PTH during lactation (3.47 +/- 0.38 pmol/L (32.72 +/- 3.58 pg/mL) versus 2.11 +/- 0.35 pmol/L (19.90 +/- 3.30 pg/mL), p = 0.02). Lippuner et al. [44] used a detection limit of 0.3 pmol/L (2.83 pg/mL), as opposed to 2.0 pmol/L (18.86 pg/mL) used by Grill et al. [41]. A similar finding was noted, with prolactin, being significantly higher during lactation than after weaning (39 +/- 10 μg/L (39 +/- 10 ng/mL) versus 13 +/- 9 μg/L (13 +/- 9 ng/mL), p = 0.018). Of note, the authors demonstrate a significant, positive correlation between prolactin and PTHrP during lactation (r = 0.8, p = 0.002), but note that there is no correlation during weaning. Lippuner et al. [44] demonstrated increased corrected calcium during lactation (2.18 +/- 0.02 mmol/L (8.74 +/- 0.08 mg/dL) versus 2.31 +/- 0.02 mmol/L (9.26 +/- 0.08 mg/dL), p = 0.003).

At two to three days postpartum, Dobnig et al. [43] demonstrated that PTHrP is positively correlated with ionized calcium (r = 0.32, p = 0.008) and negatively correlated with PTH (r = - 0.31, p < 0.01). A further finding is demonstrated by the authors, noting a negative correlation between intact PTH and ionized calcium (r = - 0.24, p = 0.03) at two to three days.

As demonstrated in the study by Segal et al. [23], there is an apparent ‘lactational osteoporosis’, which appears to reverse over time. This is not specific to hypoparathyroid women, as it was demonstrated by both More et al. [45] and Iwamoto et al. [46] in non-hypoparathyroid women, noting low bone mineral density during lactation, demonstrating a gradual normalization of bone turnover and improvement in bone mineral density once weaning had terminated.

Sowers et al. [47] demonstrated a temporary reduction in skeletal mass in relation to mothers who lactate, noting a significant, negative association between serum PTHrP and bone mineral density at the spine (p < 0.001) and femoral neck (p < 0.001). As noted by Ardawi et al. [42], PTHrP demonstrates a significant, positive correlation up to term with both alkaline phosphatase (r = 0.051, p < 0.001) and osteocalcin (r = 0.023, p < 0.05), the former finding being replicated by Gallacher et al. [36] (r = 0.44, p < 0.005). Lippuner et al. [44] demonstrated alkaline phosphatase to be significantly greater during lactation compared to weaning (74 +/- 7.1 IU/L versus 52.6 +/- 6.9 IU/L, p = 0.03). Gallacher et al. [36] suggested the elevated alkaline phosphatase during pregnancy may be of placental origin, reinforcing the idea that PTHrP is predominantly produced by the placenta during pregnancy, and there is a ‘transition’ to mammary gland secretion during lactation.

In the first six months of the study performed by Kalkwarf et al. [37], the authors note greater levels of markers of bone resorption (urinary cyclic adenosine monophosphate (cAMP) and urinary deoxypyridinoline) amongst the lactating women from baseline to the first six months. Conversely, markers of bone formation (carboxyterminal propeptide of type I procollagen (PICP) and osteocalcin) appear to be lower at baseline, increasing as lactation progresses (and weaning commences). Anai et al. [18] further demonstrated a pattern of increasing urinary cAMP from pregnancy (1.09 +/- 0.13 micromol/day) through lactation (1.97 +/- 0.23 micromol/day), which subsequently decreases post-weaning (1.28 +/- 0.09 micromol/day). The authors demonstrated urinary cAMP to be significantly greater during lactation compared to pregnancy (p < 0.0001) and post-weaning (p < 0.0001).

Hypercalcemia

In the cases reported under the ‘results’ section of the review, it is important to note that not all authors were able to measure serum PTHrP. In the report posed by Shomali et al. [17], however, the authors present raised measurements of PTHrP during lactation, decreasing as supplements required reintroduction.

Anai et al. [18] demonstrated, in their patient (second pregnancy), measurable PTHrP throughout pregnancy (1.09 +/- 0.11 pmol/L) (10.28 +/- 1.04 pg/mL), lactation (1.23 +/- 0.15 pmol/L) (11.60 +/- 1.41 pg/mL), and post-weaning (0.96 +/- 0.1 pmol/L) (9.05 +/- 0.94 pg/mL). The authors demonstrated PTHrP to be significantly greater during lactation compared to pregnancy (p < 0.05) and post-weaning (p < 0.01). Mather et al. [19] noted, in their case study, undetectable PTH throughout 72 weeks of follow-up reciprocated by a consistently elevated (detectable) PTHrP.

In the first patient described by Yasumatsu et al. [20], it is noted that, alongside her hypercalcemia at seven days postpartum, detectable PTHrP was concurrently documented. Similarly, in the report by Segal et al. [23], upon presentation of their patient, PTHrP was noted to be elevated alongside calcium; the authors further demonstrate PTHrP becoming undetectable as the serum calcium begins to decrease.

The phenomenon of apparent PTH-independent hypercalcemia in pregnant and lactating females (as noted in the above series) has furthermore been replicated across numerous case reports consisting of patients without a diagnosis of hypoparathyroidism. Marx et al. [48] presented a case of hypercalcemia (5-7 mEq/L) and suppressed PTH in an 18-year-old female in the absence of pregnancy (benign breast dysplasia); following bilateral mastectomy, serum calcium normalized (alongside PTH). There was an initial ‘drop’ in calcium likened to the description of ‘hungry bone syndrome’ by Al-Nozha et al. [24].

Van Heerden et al. [49] paralleled these aforementioned reports, presenting a 25-year-old woman with bilateral mammary hyperplasia occurring during her third pregnancy, noting hypercalcemia (3.57 mmol/L) (14.31 mg/dL) and undetectable PTH. The patient is noted to have ‘virginal hypertrophy’, requiring a bilateral mastectomy; 36 hours following surgery, serum calcium stabilized (2.42 mmol/L) (9.7 mg/dL). Additionally, immunostaining of the same patient, as described by Khosla et al. [50], demonstrated PTHrP in the breast tissue. Jackson et al. [51] presented a case of a 24-year-old female with hypercalcemia (14.3 mg/dL) noted at 24 weeks gestation with undetectable PTH, and bilateral mammary hyperplasia (requiring a bilateral mastectomy); postoperatively, the serum calcium normalized. Modarressi et al. [52] have described a similar occurrence of ‘gestational gigantomastia’ and hypercalcemia (14 mg/dL), whereby the authors demonstrate suppressed PTH and elevated PTHrP; rather than breast reduction, the patient requested termination of pregnancy, which subsequently normalized calcium, PTH, and PTHrP. Moreover, Barry et al. [53] presented a non-pregnant 15-year-old female with ‘virginal hypertrophy’ of the breasts, demonstrating elevated PTHrP, suppressed PTH, and hypercalcemia (16 mg/dL), who was managed with saline diuresis and reduction mammoplasty.

Siskind et al. [54] presented a multiparous female with a medullary sponge kidney who developed hypercalcemia during all five postpartum periods (lactation), with normalization of calcium upon weaning. Of note, the medullary sponge kidney is associated with hyperparathyroidism, but it was demonstrated that this patient did not have such a diagnosis, and the workup depicted low levels of PTH. A bone biopsy had been completed, describing consistency with excess PTH, which is presumed to be due to PTHrP (not measured).

Sato [55] described a case of a 28-year-old female without a diagnosis of hypoparathyroidism who developed symptomatic postpartum hypercalcemia (corrected calcium 19.4 mg/dL) (in the absence of lactation), alongside suppressed PTH and elevated PTHrP; the author suggested the cause of the hypercalcemia is likely due to PTHrP hypersecretion from the placenta. Whilst no measurements of calcium during pregnancy were presented by Sato [55], it is likely that they would be elevated alongside the production of PTHrP from the placenta. As this was the first day postpartum and the patient did not lactate, a placental source is the most likely origin for the PTHrP. Eller-Vainicher et al. [56] presented a 35-year-old female without hypoparathyroidism, who presents with hypercalcemia (21 mg/dL) during pregnancy at 32 weeks, alongside undetectable PTH and elevated PTHrP. The authors note serum calcium was resistant to traditional therapy with intravenous fluids. However, following the caesarean section, serum calcium freely normalized and PTHrP became readily undetectable. Authors, such as Sato [55], suggested that the PTHrP hypersecretion leading to hypercalcemia during pregnancy was of placental origin. Koren et al. [57] presented a 38-year-old female without hypoparathyroidism developing hypercalcemia during pregnancy (12.7 mg/dL), alongside suppressed PTH; the authors suggested that this is likely due to PTHrP hypersecretion from the placenta (not measured) and noted significant resistance to conventional treatment (loop diuretics, isotonic saline, and calcitonin), requiring management with pamidronate (levels normalized postpartum). Of note, the authors do not mention whether the patient breastfed following delivery.

Lepre et al. [58] described two cases of hypercalcemia in late gestation and lactation in a patient without hypoparathyroidism. The first case depicts a 34-year-old female, presenting with hypercalcemia towards the end of gestation (11.9-14.0 mg/dL at weeks 35 and 38), alongside suppressed PTH and elevated PTHrP. The authors noted that serum calcium was resistant to treatment with prednisone, but resolved by six months postpartum. The patient presents two years later with hypercalcemia throughout pregnancy alongside elevated PTHrP, both of which remained elevated for several months during the postpartum period (lactation). Lescuyer et al. [59] presented a 29-year-old female (without a diagnosis of hypoparathyroidism) at three months postpartum (lactating) with hypercalcemia (3.09 mmol/L) (12.38 mg/dL), suppressed PTH and elevated PTHrP; the authors note upon weaning serum calcium normalized (2.34 mmol/L) (9.38 mg/dL) and PTHrP became undetectable. Shi et al. [60] presented a 27-year-old postpartum, lactating female with no known history of hypoparathyroidism, demonstrating suppressed PTH and hypercalcemia (3.45 mmol/L) (13.83 mg/dL); the authors did not measure PTHrP but suggested that this is the most likely explanation after ruling out other causes.

In a similar manner to the case report by Segal et al. [23], Kebapci et al. [61] presented a case of a patient without hypoparathyroidism, presenting two months postpartum (during lactation) with an L5 vertebral fracture. Upon further assessment, she was noted to demonstrate hypercalcemia (10.24 mg/dL), suppressed PTH, significant osteoporosis, and elevated alkaline phosphatase; the presentation is presumed to be due to PTHrP.

Breslau et al. [62] described two patients (covering four pregnancies) with pseudohypoparathyroidism, whereby serum calcium normalized during pregnancy (alongside a decrease in PTH and an increase in 1,25-dihydroxyvitamin D). The authors noted that, following delivery, serum calcium and 1,25-dihydroxyvitamin D decreased, alongside an increase in PTH. The authors do not describe if the patients breastfed, and therefore it is unclear how patients with pseudohypoparathyroidism would present during lactation. Kovacs et al. [33] suggested that the placental sources of 1,25-dihydroxyvitamin D are lost after birth, and the kidneys will remain resistant to PTHrP; therefore, calcitriol supplements are likely to return to pre-pregnancy levels.

Prolactin

Prolactin is a hormone involved in lactation and is released from the lactotrophs in the anterior pituitary in response to a myriad of stimuli (predominantly nipple suckling). As demonstrated by Sowers et al. [47], there is a positive correlation between PTHrP and prolactin; PTHrP is present in the serum at levels significantly greater in lactating mothers (as is prolactin) compared to those who bottle-feed (p < 0.001). Ardawi et al. [42] furthermore noted a significant, positive correlation between PTHrP and prolactin (r = 0.41, p < 0.05) throughout pregnancy. Sowers et al. [47] noted that PTHrP was detectable more than 50% of the time (above 0.35 pmol/L (3.30 pg/mL)) in those breastfeeding, compared to less than 6% of the time in those bottle-feeding. Additionally, Cundy et al. [11] demonstrated that a decline in serum prolactin (during weaning) is mirrored by a decline in serum PTHrP (countered by an increase in serum E2). As demonstrated by Sowers et al. [47], breast-feeding women had a significantly lower serum E2 (p < 0.001); conversely, patients with higher E2 had lower prolactin levels (similarly, those with undetectable PTHrP demonstrated greater E2 levels).

It should be noted that elevated PTHrP has been documented in other cases of hyperprolactinemia, such as that with prolactinomas (albeit levels of PTHrP were not as elevated as those in lactating women) [19]. In rat studies, as noted by Sowers et al. [47], there is a temporal relationship with regard to prolactin and the expression of PTHrP messenger RNA (mRNA). Winter et al. 30] demonstrated a case of hypercalcemia during pregnancy (from elevated PTHrP), which normalized following the administration of bromocriptine (dopamine (D2) receptor agonist). Greer et al. [63] demonstrated that nursing twins (as opposed to a singleton pregnancy) leads to greater prolactin and ultimately serum calcium (in addition to 1,25-dihydroxyvitamin D and calcitonin). However, this was not replicated in the study by Nakayama et al. [64].

Kovacs et al. [65] analyzed 33 lactating women and 16 patients with pituitary adenoma, with both cohorts and a control group. In the lactating group, mean calcium was greater in lactating women compared to controls (2.39 +/- 0.01 mmol/L (9.58 +/- 0.04 mg/dL) versus 2.35 +/- 0.01 mmol/L (9.42 +/- 0.04 mg/dL), p < 0.01). Moreover, mean PTH was significantly lower (2.49 +/- 0.24 pmol/L (23.48 +/- 2.26 pg/mL) versus 3.17 +/- 0.23 pmol/L (29.90 +/- 2.17 pg/mL), p < 0.04). Conversely, mean PTHrP was greater in the lactating women compared to controls (0.93 +/- 0.08 pmol/L (8.77 +/- 0.75 pg/mL) versus 0.38 +/- 0.04 pmol/L (3.59 +/- 0.38 pg/mL), p < 0.001). The authors demonstrated a negative correlation between prolactin and PTH (r = - 0.41, p < 0.02) and a positive correlation between prolactin and PTHrP (r = 0.38, p < 0.05). When analyzing the adenoma cohort, however, there was no significant difference between the subjects and controls when comparing mean calcium and PTH. However, mean PTHrP was greater in the adenoma group (0.75 +/- 0.10 pmol/L (7.07 +/- 0.94 pg/mL) versus 0.39 +/- 0.07 pmol/L (3.68 +/- 0.66 pg/mL), p < 0.006), being detectable in 14 of the 16 subjects. Moreover, in a manner similar to Winter et al. [30], the elevated PTHrP in the adenoma group significantly decreased with the introduction of therapy to normalize prolactin (p < 0.03).

Of further peculiarity, serum prolactin appears to not only be intricately associated with serum PTHrP but also 1,25-hydroxyvitamin D3 (active vitamin D). Following the work by Cundy et al. [11], the apparent ‘half-life’ of active vitamin D appears to be prolonged in the presence of elevated serum prolactin. This is described as an inverse correlation between serum prolactin and calcitriol requirements during the first year postpartum (p < 0.001).

Vitamin D

Cundy et al. [11] attempted to further analyze and understand the prolongation of the half-life of 1,25-hydroxyvitamin D3 by intentionally inducing hypercalcemia at 13 months postpartum and by comparing the rate of decline of calcium (and vitamin D metabolites) in lactating and non-lactating physiological states. The authors demonstrated that, during lactation when the patient had calcitriol toxicity requiring discontinuation (peak calcium 3.32 mmol/L (13.31 mg/dL)), there was a half-life of 27 days once calcitriol was stopped; this contrasts with the intentional induction of hypercalcemia in a non-lactational state (peak serum calcium 3.08 mmol/L (12.34 mg/dL)), with a half-life of only three days (p < 0.0001). Additionally, Cundy et al. [11] noted that 25-OHD did not change in a significant manner during calcitriol treatment (including pregnancy and lactation), suggesting there is no relationship between 1,25-hydroxyvitamin D3 and 25-OHD. Whilst 24,25(OHD)2D3 remained stable during intentionally induced hypercalcemia, during lactation, 24,25(OHD)2D3 mirrored the rise and fall of 1,25-hydroxyvitamin D3, demonstrating a positive correlation in the early postpartum (p < 0.05). Kovacs et al. [33] noted that in non-hypoparathyroid women (in the postpartum period) both free and bound 1,25-dihydroxyvitamin D fall to normal (remaining so during both lactation and weaning, potentially rising thereafter). Greer et al. [63] suggested that serum 1,25-dihydroxyvitamin D is greater in those nursing twins compared to singletons.

The correlation between the prolongation of active vitamin D and prolactin is ill-defined, albeit three strong hypotheses are currently understood to be partly correct: delayed clearance, PTHrP-mediated, and prolactin-induced. The most straightforward explanation for a prolongation of the half-life of active vitamin D and prolactin would be that there is a reduction in the clearance of the former (as opposed to enhanced endogenous production). This hypothesis, while simple, is not accepted by many, as it is evident that serum 1,25-hydroxyvitamin D3 levels may continue to rise following discontinuation of calcitriol supplementation [11].

PTHrP demonstrates similarity in structure to PTH (the first 34 amino acids of PTHrP are similar in structure to PTH), acting upon the same cell receptors [38]. Anecdotal evidence suggests that PTHrP may directly stimulate the enzyme 1-α-hydroxylase (present within the maternal kidney). However, it is believed that PTHrP is at best a weak stimulator of this enzyme, a finding demonstrated in human subjects by Horwitz et al. [66], who points out the notion that serum levels of 1,25-hydroxyvitamin D3 are often depleted in patients with humoral hypercalcemia of malignancy.

Prolactin has similarly been suggested to be able to stimulate the enzyme 1-α-hydroxylase (CYP27B1) within the maternal kidney. In both chick and rat studies, this theory has been demonstrated on multiple occasions [67-69]. As reported by Ajibade et al. [70], prolactin demonstrates a direct effect on the transcription of the 1-α-hydroxylase gene. Furthermore, in lactating rats, the introduction of bromocriptine was demonstrated to lead to a reduction in serum 1,25-hydroxyvitamin D3 [71]. In addition to the proposed effect of prolactin upon 1-α-hydroxylase, it has also been noted by Ajibade et al. [70] that prolactin may directly up-regulate the expression of transient receptor potential vanilloid subfamily member 6 (TRPV6) mRNA, which is responsible for calcium absorption in the intestine. Rude et al. [10] further suggested that prolactin may directly increase fractional renal calcium reabsorption.

Markestad et al. [9] presented a patient with hypoparathyroidism developing hypercalcemia during lactation and compared the metabolism of vitamin D against three arms of controls without hypoparathyroidism: non-pregnant (n = 17), pregnant (at delivery) (n = 22,) and during days three and 16 of lactation (n = 8). The authors demonstrate the plasma concentrations of 1,25-dihydroxyvitamin D to be greater in pregnancy at term (203 pmol/L) (1,914.29 pg/mL) compared to the non-pregnant state (86 pmol/L) (810.98 pg/mL) (p < 0.0005). Additionally, the postpartum measurements on days three (57 pmol/L) (537.51 pg/mL) and 16 (62 pmol/L) (584.66 pg/mL) were significantly lower compared to those of the non-pregnant women (p < 0.01). As noted in the case report by Markestad et al. [9], the serum levels of 1,25-dihydroxyvitamin D were similar to the controls on day three postpartum (lactation), but markedly increased by two weeks. The authors note that their patient required discontinuation of alfacalcidol, whereby serum levels of 1,25-dihydroxyvitamin D returned to normal within three days (and when the patient required high dosages of vitamin D2 later, serum 1,25-dihydroxyvitamin D remained normal in the presence of moderate hypercalcemia when studied at 16 weeks postpartum). The results by Mather et al. [19] demonstrate a reduction in serum 1,25-dihydroxyvitamin D upon discontinuation of calcitriol supplementation. However, they note that the levels of 1,25-dihydroxyvitamin D remained within the reference range. Rude et al. [10] noted a similar finding of 1,25-dihydroxyvitamin D remaining within the reference range (albeit at the lower end of normal) upon discontinuation of calcitriol. Markestad et al. [9] noted that the serum 25-OHD appeared to remain unchanged in the postpartum period, with 24,25-(OH)2D increasing in all lactating women, measured at day three (2.7 nmol/L) (1.08 ng/mL) and day 16 (3.7 nmol/L) (1.48 ng/mL). Whilst 25-OHD in the controls did not change significantly during the first 16 days postpartum, there was a significant increase in their patient. There is no clear explanation for this finding, as 25-OHD continued to rise despite a drop in calcium and 1,25-dihydroxyvitamin D following discontinuation of alfacalcidol; it is speculated that this is due to mobilization of vitamin D from adipose tissue from a catabolic state. Moreover, in a manner similar to the control group, 24,25(OH)2D increased postpartum, as noted by Markestad et al. [9]. Markestad et al. [9] therefore concluded that, whilst there may be an increased requirement for vitamin D during pregnancy, there is a rapid reduction in requirements during lactation, in both healthy and hypoparathyroid women; this is partially explained by the increase in 24,25(OH)2D in the postpartum period, suggesting the reduced requirement for vitamin D-mediated calcium absorption (and hence reduced synthesis of 1,25-dihydroxyvitamin D). As demonstrated by Breslau et al. [62] and Zerwekh et al. [72], during pregnancy there is evidence that increased serum 1,25-dihydroxyvitamin D is produced from the placenta. However, this does not explain the role of decreased calcitriol requirements during the postpartum period.

Hypocalcemia

One should be aware that the converse has been described during both gestation and the postpartum periods, whereby there is an apparent ‘worsening’ of serum calcium (hypocalcemia). The case report posed by Sweeney et al. [21] differs from other cases described, as the patient initially presented with postpartum hypocalcemia (6.4 mg/dL), requiring management with calcium gluconate. The authors suggest there is a ‘window of transition’ between PTHrP production from the placenta and mammary tissue, which may have caused the patient to develop hypocalcemia (lactation did not commence until day two postpartum). Ferguson II et al. [73] provided some insight into this ‘window period’ by demonstrating that the expression of PTHrP is acutely decreased in the amnion in humans at the onset of labour (p < 0.001). Moreover, following fetal membrane rupture, PTHrP mRNA in the amnion was significantly decreased by 78% (p < 0.0001), alongside PTHrP immunoreactivity (p < 0.01) and bioactivity (p < 0.03). A report posed by Lepre et al. [58] further demonstrates a reduction in PTHrP following delivery, followed by an increase during lactation. It is important to note, however, that the patient described by Sweeney et al. [21] received magnesium sulphate for pre-eclampsia; magnesium is a known cause of hypocalcemia (as demonstrated in the case report by Krysiak et al. [22]) due to inhibition of the tubular reabsorption of calcium (magnesium further inhibits the release of PTH, but the secretion of this latter hormone is already deficient in the patient).

Al-Nozha et al. [24] further suggested that the release of prostaglandin E2 during the process of parturition may enhance the mobilization of calcium from bodily stores; the authors also suggested that a sudden ‘drop’ in this hormone may lead to postpartum hypocalcemia. Blickstein et al. [74] reflected this suggestion, demonstrating a patient with hypoparathyroidism requiring induction with prostaglandin E2 vaginal tablets and depicting a course of postpartum hypocalcemia (7 mg/dL). This is again demonstrated by Shah et al. [25], whereby a hypoparathyroid patient develops symptomatic hypocalcemia after induction (two administrations) with prostaglandin E2 gel.

Eller-Vainicher et al. [56] presented a case of placental PTHrP hypersecretion, leading to hypercalcemia (21 mg/dL) at 32 weeks of gestation (alongside suppressed PTH and elevated PTHrP). The authors noted that, following the caesarean section, serum calcium initially normalized, followed by a decrease, countered with increasing PTH over the subsequent days. Eller-Vainicher et al. [56] described this as a clinical picture similar to the hungry bone syndrome.

Durst et al. [75] described a 21-year-old female with idiopathic hypoparathyroidism at five days postpartum demonstrating serum calcium of 0.83 mmol/L, which required calcium (1.2 g) and active vitamin D (0.25 μg) daily. The authors noted serum calcium readily stabilized with supplements as lactation progressed over the ensuing eight months. However, hypocalcemia reappeared when weaning commenced (at which point higher dosages were required: 2.4 g of calcium and 0.5 μg of active vitamin D).

Bernstein et al. [76] depicted a 17-year-old female with idiopathic hypoparathyroidism; the authors noted that the patient suppressed lactation (after nine days) with stilboestrol. Around one month later, the patient presented with hypocalcemic tetany, requiring 100,000 IU of calciferol (reduced to 50,000 IU upon discharge) and 12 g of calcium lactate per day (discontinued upon discharge). However, the patient was readmitted one month later with another episode of symptomatic hypocalcemia, requiring alterations with dihydrotachysterol and calcium lactate. Similarly, Tordjman et al. [77] noted a case of asymptomatic hypoparathyroidism in a 25-year-old mother during pregnancy; the patient presented with symptomatic hypocalcemia (5 mg/dL) shortly after a caesarean section, requiring hospitalization for 17 days and various calcium-vitamin D therapeutic combinations; of note, it appears that this patient did not lactate, in keeping with ‘nadir’ following PTHrP-placental removal.

Harrad et al. [78] presented a 28-year-old female at eight months following a caesarean section, during which time, the patient had been lactating; the authors demonstrated hypocalcemia on multiple occasions (1.45-1.54 mmol/L) (5.81-6.17 mg/dL), suppressed PTH, and a history of four grand-mal seizures, from which she was diagnosed with hypoparathyroidism and required intravenous calcium gluconate, followed by 2 g of daily cholecalciferol and a high-calcium diet. The authors curiously demonstrate, however, that the seizures only occurred towards the end of prolonged lactation, from which it is believed calcium loss was increased.

Callies et al. [79] presented a 29-year-old female, demonstrating labile elemental calcium and calcitriol requirements throughout pregnancy. During the lactational period, the authors noted that the patient presented with nocturnal tetany; this was despite 1 μg of calcitriol and between 4 and 6 g of calcium per day. During the patient’s second pregnancy, the authors note she required a higher dosage of calcitriol (0.75 μg versus 0.25 to 0.50 μg) and calcium (3 g per day) during the lactation period compared to the pregnancy period.

Jabbar et al. [80] discussed a 26-year-old female, whereby both of her pregnancies ‘unmasked’ underlying hypoparathyroidism; in the first pregnancy, her serum calcium was 5.7 mg/dL, returning to normal after birth. In the second pregnancy, serum calcium was 4.5 mg/dL requiring treatment with calcium gluconate, calcidiol (1 μg per day), and oral calcium carbonate. The authors note that the patient did not attend follow-up; however, after being contacted by the staff eight weeks postpartum, the patient noted that she was not symptomatic and had discontinued her supplements and that her serum calcium had normalized. Unfortunately, the authors did not mention whether the patient was breastfeeding, limiting the interpretation of this report.

Kurzel et al. [81] presented a case of hypocalcemia in a patient with hypoparathyroidism during pregnancy (requiring up to 15 g of calcium and 0.25 μg of calcitriol), for which 50 mg of hydrochlorothiazide was added to limit hypercalciuria, allowing stabilization of her serum calcium. Following delivery, however, the calcium supplement was reduced to 6 g, and she was advised not to breastfeed. Salle et al. [82] presented a 30-year-old female with hypoparathyroidism requiring increasing dosages of calcitriol (0.5-2.0 μg daily) during her pregnancy to maintain stable serum calcium levels; of note, however, that there is no mention of lactation during the postpartum period.

Recommendations

Both the hypoparathyroid mother and her infant should be carefully monitored to detect abnormal calcium levels [12,13]. The monitoring, as noted by Khan et al. [83], should begin during pregnancy, as there is no reliable predictor as to when the patient’s calcitriol requirements are likely to change - calcitriol should not be inappropriately continued (or terminated) during pregnancy and lactation unless there is appropriate reasoning. Inappropriate discontinuation during pregnancy may lead to hypocalcemia in the mother (and hyperplasia of the fetal parathyroid glands). Conversely, inappropriate continuation may lead to hypercalcemia in the mother (and suppression of the fetal parathyroid glands). Tsourdi et al. [84] recommended monitoring calcium every four to six weeks during lactation to ensure that normocalcemia is present and to wean as appropriate. Khan et al. [83] recommended weekly albumin-corrected (or ionized) calcium levels during the first month postpartum, with a target of achieving calcium within the low-normal reference range (in a manner similar to a non-breastfeeding female). All women with hypoparathyroidism during gestation should be educated regarding the need to be closely assessed for signs and symptoms of either hypercalcemia or hypocalcemia and to be aware that a change in dosage may be required; stratification should occur early on regarding patients who are likely to breastfeed. Furthermore, records during prior pregnancies should be obtained in women with hypoparathyroidism to assess when hypercalcemia (or hypocalcemia) occurred during a previous pregnancy (or postpartum period) and to consider a proactive dosage reduction in supplementation should hypercalcemia have occurred in a prior pregnancy. As a final consideration, in patients who do require active vitamin D supplementation during gestation and lactation, Tsourdi et al. [84] suggested calcitriol due to its shorter half-life (more rapid recovery from hypercalcemia).

Strengths and limitations

A major strength of this article includes the enhanced awareness of a niche endocrine phenomenon, for which access will be available to all healthcare professionals who may encounter a female patient with hypoparathyroidism. Moreover, this article provides an up-to-date review of the available literature depicted by various authors globally. As with all articles, however, limitations must be highlighted; in this case, it is important to note that a great proportion of females with hypoparathyroidism may not require dosage adjustments during either gestation or lactation, with these apparently ‘normal’ cases unlikely to be reported upon (therefore a true estimation of the proportion of patients with hypoparathyroidism who develop hypercalcemia during lactation cannot be calculated). Further risks include selection bias and misinterpretation of data from articles published in German, Japanese, and French (the former two requiring external translation). Moreover, as there are multiple underlying causes of hypoparathyroidism (as depicted in Table 1), a question that remains unanswered is whether the apparent improvement in serum calcium during lactation is more prominent in certain forms of hypoparathyroidism than others. Sadeghi-Nejad et al. [8] suggested that patients with surgical hypoparathyroidism (post-thyroidectomy) may be at an increased risk due to the lack of calcitonin (a hormone that decreases serum calcium). However, this has not been studied in humans (in both rat and goat studies, calcitonin-deficient subjects demonstrated more bone mineral loss (and rises in serum calcium) compared to controls)) [33,85-87]. A further clinical area that remains unanswered is posed by Marcucci et al. [27], who demonstrate the hypercalcemia noted during the lactational period may be confounded by an increase in dietary calcium; this has not been adequately assessed within the literature. Another point requiring clarification is the association between peripheral (non-neuronal) serotonin and PTHrP production [88]; in animal models, the absence of tryptophan hydroxylase may present with lactation-associated hypocalcemia, a finding which was reversed by Laporta et al. [89] with daily 5-hydroxytryptamine injections. Furthermore, as noted by Winter et al. [38], an intact oxytocin axis appears to be required for bone resorption during lactation, findings that have yet to be adequately assessed in human subjects. As noted by Kovacs [90], during pregnancy, there is an ‘artefactual’ decline in total calcium noted by the increased intravascular volume (hemodilution) and depleted albumin; this must be addressed in future studies, as many authors have not accounted for this finding (nor has the ionized calcium been measured), leading to unnecessary supplementation with calcium and/or calcitriol. A final consideration, as noted by Tsourdi and Anastasilakis [84], is that calcitriol has a shorter half-life compared to other active vitamin D supplements; in case reports in which other supplements have been used, it will take longer for the hypercalcemia to resolve.

## Conclusions

This article provides a scoping review of the literature pertaining to the phenomenon of apparent improvement in serum calcium control during late gestation and lactation in females with hypoparathyroidism. In addition, the pathophysiology of this concept is reviewed, which is paralleled to case series whereby this occurrence has been described in patients without a pre-existent diagnosis of hypoparathyroidism. It is very important that this concept is anticipated to avoid detrimental, maternal hypercalcemia during gestation and postpartum; conversely, however, as demonstrated, this is not a universal finding, with some patients demonstrating a worsening in their serum calcium control (requiring increased supplementation). For this reason, an individualized approach with close monitoring is essential when treating hypoparathyroid females during gestation and the postpartum period.

## References

[1] Kafetzis ID, Diamantopoulos A, Christakis I, Leoutsakos B (2011). The history of the parathyroid glands. Hormones (Athens).

[2] Kalra S, Baruah MP, Sahay R, Sawhney K (2013). The history of parathyroid endocrinology. Indian J Endocrinol Metab.

[3] Bilezikian JP, Khan A, Potts JT Jr (2011). Hypoparathyroidism in the adult: epidemiology, diagnosis, pathophysiology, target-organ involvement, treatment, and challenges for future research. J Bone Miner Res.

[4] Goltzman D (2022). Hypoparathyroidism. UpToDate.

[5] Richa CG, Issa AI, Echtay AS, El Rawas MS (2018). Idiopathic hypoparathyroidism and severe hypocalcemia in pregnancy. Case Rep Endocrinol.

[6] Hatswell BL, Allan CA, Teng J (2015). Management of hypoparathyroidism in pregnancy and lactation - a report of 10 cases. Bone Rep.

[7] Wright AD, Joplin GF, Dixon HG (1969). Post-partum hypercalcaemia in treated hypoparathyroidism. Br Med J.

[8] Sadeghi-Nejad A, Wolfsdorf JI, Senior B (1980). Hypoparathyroidism and pregnancy treatment with calcitriol. JAMA.

[9] Markestad T, Ulstein M, Bassoe HH, Aksnes L, Aarskog D (1983). Vitamin D metabolism in normal and hypoparathyroid pregnancy and lactation. Case report. Br J Obstet Gynaecol.

[10] Rude RK, Haussler MR, Singer FR (1984). Postpartum resolution of hypocalcemia in a lactating hypoparathyroid patient. Endocrinol Jpn.

[11] Cundy T, Haining SA, Guilland-Cumming DF, Butler J, Kanis JA (1987). Remission of hypoparathyroidism during lactation: evidence for a physiological role for prolactin in the regulation of vitamin D metabolism. Clin Endocrinol (Oxf).

[12] Caplan RH, Beguin EA (1990). Hypercalcemia in a calcitriol-treated hypoparathyroid woman during lactation. Obstet Gynecol.

[13] Caplan RH, Wickus GG (1993). Reduced calcitriol requirements for treating hypoparathyroidism during lactation. A case report. J Reprod Med.

[14] Höper K, Pavel M, Dörr HG, Kändler C, Kruse K, Wildt L, Hensen J (1994). [Calcitriol administration during pregnancy in a partial DiGeorge anomaly]. Dtsch Med Wochenschr.

[15] Kennedy Kennedy, L. and Arce, K. (2016 (2022). An unusual case of hypercalcemia. https://consultqd.clevelandclinic.org/unusual-case-hypercalcemia-2/.

[16] Cathébras P, Cartry O, Sassolas G, Rousset H (1996). Hypercalcemia in two treated hypoparathyroid lactating women. Rev Med Interne.

[17] Shomali ME, Ross DS (1999). Hypercalcemia in a woman with hypoparathyroidism associated with increased parathyroid hormone-related protein during lactation. Endocr Pract.

[18] Anai T, Tomiyasu T, Takai N, Miyakawa I (1999). Remission of idiopathic hypoparathyroidism during lactation: a case report. J Obstet Gynaecol Res.

[19] Mather KJ, Chik CL, Corenblum B (1999). Maintenance of serum calcium by parathyroid hormone-related peptide during lactation in a hypoparathyroid patient. J Clin Endocrinol Metab.

[20] Yasumatsu R, Nakashima T, Kuratomi Y, Komiyama S (2002). [Postpartum hypercalcemia in a patient with previous thyroid carcinoma: a report of 2 cases]. Nihon Jibiinkoka Gakkai Kaiho.

[21] Sweeney LL, Malabanan AO, Rosen H (2010). Decreased calcitriol requirement during pregnancy and lactation with a window of increased requirement immediately post partum. Endocr Pract.

[22] Krysiak R, Kobielusz-Gembala I, Okopien B (2011). Hypoparathyroidism in pregnancy. Gynecol Endocrinol.

[23] Segal E, Hochberg I, Weisman Y, Ish-Shalom S (2011). Severe postpartum osteoporosis with increased PTHrP during lactation in a patient after total thyroidectomy and parathyroidectomy. Osteoporos Int.

[24] Al Nozha OM, Malakzadeh-Shirvani P (2013). Calcium homeostasis in a patient with hypoparathyroidism during pregnancy, lactation and menstruation. J Taibah Univ Med Sci.

[25] Shah KH, Bhat S, Shetty S, Umakanth S (2015). Hypoparathyroidism in pregnancy. BMJ Case Rep.

[26] Dixon J, Miller S (2018). Successful pregnancies and reduced treatment requirement while breast feeding in a patient with congenital hypoparathyroidism due to homozygous c.68C>A null parathyroid hormone gene mutation. BMJ Case Rep.

[27] Marcucci G, Altieri P, Benvenga S (2021). Hypoparathyroidism and pseudohypoparathyroidism in pregnancy: an Italian retrospective observational study. Orphanet J Rare Dis.

[28] Hartogsohn EA, Khan AA, Kjaersulf LU, Sikjaer T, Hussain S, Rejnmark L (2020). Changes in treatment needs of hypoparathyroidism during pregnancy and lactation: a case series. Clin Endocrinol (Oxf).

[29] Wang JJ, Wang O, Wang YB (2021). Changes in serum calcium and treatment of hypoparathyroidism during pregnancy and lactation: a single-center case series. J Clin Endocrinol Metab.

[30] Winter EM, Appelman-Dijkstra NM (2017). Parathyroid hormone-related protein-induced hypercalcemia of pregnancy successfully reversed by a dopamine agonist. J Clin Endocrinol Metab.

[31] van Wingerden JJ (2009). Gigantomastia--definition and association with hypercalcaemia. J Plast Reconstr Aesthet Surg.

[32] Ali DS, Dandurand K, Khan AA (2021). Hypoparathyroidism in pregnancy and lactation: current approach to diagnosis and management. J Clin Med.

[33] Kovacs CS, Kronenberg HM (1997). Maternal-fetal calcium and bone metabolism during pregnancy, puerperium and lactation. Endocr Rev.

[34] Lebrun B, De Block C, Jacquemyn Y (2020). Hypocalcemia after thyroidectomy and parathyroidectomy in a pregnant woman. Endocrinology.

[35] Kovacs CS, Lanske B, Hunzelman JL, Guo J, Karaplis AC, Kronenberg HM (1996). Parathyroid hormone-related peptide (PTHrP) regulates fetal-placental calcium transport through a receptor distinct from the PTH/PTHrP receptor. Proc Natl Acad Sci U S A.

[36] Gallacher SJ, Fraser WD, Owens OJ (1994). Changes in calciotrophic hormones and biochemical markers of bone turnover in normal human pregnancy. Eur J Endocrinol.

[37] Kalkwarf HJ, Specker BL, Ho M (1999). Effects of calcium supplementation on calcium homeostasis and bone turnover in lactating women. J Clin Endocrinol Metab.

[38] Winter EM, Ireland A, Butterfield NC (2020). Pregnancy and lactation, a challenge for the skeleton. Endocr Connect.

[39] Kovacs CS (2016). Maternal mineral and bone metabolism during pregnancy, lactation, and post-weaning recovery. Physiol Rev.

[40] Kalkwarf HJ, Specker BL, Heubi JE, Vieira NE, Yergey AL (1996). Intestinal calcium absorption of women during lactation after weaning. Am J Clin Nutr.

[41] Grill V, Hillary J, Ho PM (1992). Parathyroid hormone-related protein: a possible endocrine function in lactation. Clin Endocrinol (Oxf).

[42] Ardawi MS, Nasrat HA, BA'Aqueel HS (1997). Calcium-regulating hormones and parathyroid hormone-related peptide in normal human pregnancy and postpartum: a longitudinal study. Eur J Endocrinol.

[43] Dobnig H, Kainer F, Stepan V (1995). Elevated parathyroid hormone-related peptide levels after human gestation: relationship to change in bone and mineral metabolism. J Clin Endocrinol Metab.

[44] Lippuner K, Zehnder HJ, Casez JP, Takkinen R, Jaeger P (1996). PTH-related protein is released into the mother's bloodstream during lactation: evidence for beneficial effects on maternal calcium-phosphate metabolism. J Bone Miner Res.

[45] More C, Bettembuk P, Bhattoa HP, Balogh A (2001). The effects of pregnancy and lactation on bone mineral density. Osteoporos Int.

[46] Iwamoto J, Sato Y, Uzawa M, Matsumoto H (2012). Five-year follow-up of a woman with pregnancy and lactation-associated osteoporosis and vertebral fractures. Ther Clin Risk Manag.

[47] Sowers MF, Hollis BW, Shapiro B (1996). Elevated parathyroid hormone-related peptide associated with lactation and bone density loss. JAMA.

[48] Marx SJ, Zusman RM, Umiker WO (1977). Benign breast dysplasia causing hypercalcemia. J Clin Endocrinol Metab.

[49] Van Heerden JA, Gharib H, Jackson IT (1988). Pseudohyperparathyroidism secondary to gigantic mammary hypertrophy. Arch Surg.

[50] Khosla S, van Heerden JA, Gharib H, Jackson IT, Danks J, Hayman JA, Martin TJ (1990). Parathyroid hormone-related protein and hypercalcemia secondary to massive mammary hyperplasia. N Engl J Med.

[51] Jackson IT, Saleh J, van Heerden JA (1989). Gigantic mammary hyperplasia in pregnancy associated with pseudohyperparathyroidism. Plast Reconstr Surg.

[52] Modarressi T, Levine MA, Tchou J, Khan AN (2018). Gestational gigantomastia complicated by PTHrP-mediated hypercalcemia. J Clin Endocrinol Metab.

[53] Barry F, Conte FA, Grumbach MM (1993). Massive virginal breast hypertrophy as a cause of recurrent humoral hypercalcemia due to parathyroid hormone-related protein (PTHrP) secretion. Pediatr Res.

[54] Siskind MS, Popovtzer MM (1991). Postpartum hypercalcemia in a patient with medullary sponge kidneys. Am J Kidney Dis.

[55] Sato K (2008). Hypercalcemia during pregnancy, puerperium, and lactation: review and a case report of hypercalcemic crisis after delivery due to excessive production of PTH-related protein (PTHrP) without malignancy (humoral hypercalcemia of pregnancy). Endocr J.

[56] Eller-Vainicher C, Ossola MW, Beck-Peccoz P, Chiodini I (2012). PTHrP-associated hypercalcemia of pregnancy resolved after delivery: a case report. Eur J Endocrinol.

[57] Koren R, Neeman O, Koren S, Benbassat CA (2018). Humoral hypercalcemia of pregnancy treated with bisphosphonates. Arch Endocrinol Metab.

[58] Lepre F, Grill V, Ho PW, Martin TJ (1993). Hypercalcemia in pregnancy and lactation associated with parathyroid hormone-related protein. N Engl J Med.

[59] Lescuyer S, Lanani A, Valentin C, Rondeau-Lutz M, Weber JC (2018). Hypercalcemia and PTH-rP... happy event!. Rev Med Interne.

[60] Shi W, Wang X, Yang J, Xu L, Bu R (2021). Non-parathyroid hypercalcemia during lactation: a case report. Ann Palliat Med.

[61] Kebapci N, Yorulmaz G, Akalin A (2016). Postpartum osteoporosis associated with hypercalcemia and hypoparathyroidism. Endocrine Abstracts.

[62] Breslau NA, Zerwekh JE (1986). Relationship of estrogen and pregnancy to calcium homeostasis in pseudohypoparathyroidism. J Clin Endocrinol Metab.

[63] Greer FR, Lane J, Ho M (1984). Elevated serum parathyroid hormone, calcitonin, and 1,25-dihydroxyvitamin D in lactating women nursing twins. Am J Clin Nutr.

[64] Nakayama S, Yasui T, Suto M (2011). Differences in bone metabolism between singleton pregnancy and twin pregnancy. Bone.

[65] Kovacs CS, Chik CL (1995). Hyperprolactinemia caused by lactation and pituitary adenomas is associated with altered serum calcium, phosphate, parathyroid hormone (PTH) and PTH-related peptide levels. J Clin Endocrinol Metab.

[66] Horwitz MJ, Tedesco MB, Sereika SM (2005). Continuous PTH and PTHrP infusion causes suppression of bone formation and discordant effects on 1,25(OH)2 vitamin D. J Bone Miner Res.

[67] Bikle DD, Spencer EM, Burke WH, Rost CR (1980). Prolactin but not growth hormone stimulates 1,25-dihydroxyvitamin D3 production by chick renal preparations in vitro. Endocrinology.

[68] Spanos E, Pike JW, Haussler MR (1976). Circulating 1alpha,25-dihydroxyvitamin D in the chicken: enhancement by injection of prolactin and during egg laying. Life Sci.

[69] Spanos E, Brown DJ, Stevenson JC, MacIntyre I (1981). Stimulation of 1,25-hihydroxycholecalciferol production by prolactin and related peptides in intact renal cell preparations in vitro. Biochim Bioiphys Acta.

[70] Ajibade DV, Dhawan P, Fechner AJ, Meyer MB, Pike JW, Christakos S (2010). Evidence for a role of prolactin in calcium homeostasis: regulation of intestinal transient receptor potential vanilloid type 6, intestinal calcium absorption, and the 25-hydroxyvitamin D(3) 1alpha hydroxylase gene by prolactin. Endocrinology.

[71] Robinson CJ, Spanos E, James MF (1982). Role of prolactin in vitamin D metabolism and calcium absorption during lactation in the rat. J Endocrinol.

[72] Zerwekh JE, Breslau NA (1986). Human placental production of 1 alpha,25-dihydroxyvitamin D3: biochemical characterization and production in normal subjects and patients with pseudohypoparathyroidism. J Clin Endocrinol Metab.

[73] Ferguson JE 2nd, Gorman JV, Bruns DE, Weir EC, Burtis WJ, Martin TJ, Bruns ME (1992). Abundant expression of parathyroid hormone-related protein in human amnion and its association with labor. Proc Natl Acad Sci U S A.

[74] Blickstein I, Kessler I, Lancet M (1985). Idiopathic hypoparathyroidism with gestational diabetes. Am J Obstet Gynecol.

[75] Durst R, Meirovitz A, Gross D, Kolker O, Muszkat M (2002). Post-partum hypocalcemia: idiopatic hypoparathyroidism manifested early in lactation. J Endocrinol Invest.

[76] Bernstein A (1967). Idiopathic hypoparathyroidism manifesting after lactation. Postgrad Med J.

[77] Tordjman K, Rosenthal T, Apter S (1985). Asymptomatic long-standing idiopathic hypoparathyroidism discovered following delivery of a healthy infant. Am J Med.

[78] Harrad RA, Kennedy PG (1982). Hypocalcaemia-induced epilepsy during lactation. Br Med J (Clin Res Ed).

[79] Callies F, Arlt W, Scholz HJ, Reincke M, Allolio B (1998). Management of hypoparathyroidism during pregnancy--report of twelve cases. Eur J Endocrinol.

[80] Jabbar A, Samad L, Akhter J, Khan MA (1998). Pregnancy unmasking hypoparathyroidism. J Pak Med Assoc.

[81] Kurzel RB, Hagen GA (1990). Use of thiazide diuretics to reduce the hypercalciuria of hypoparathyroidism during pregnancy. Am J Perinatol.

[82] Salle BL, Berthezene F, Glorieux FH (1981). Hypoparathyroidism during pregnancy: treatment with calcitriol. J Clin Endocrinol Metab.

[83] Khan AA, Clarke B, Rejnmark L, Brandi ML (2019). Hypoparathyroidism in pregnancy: review and evidence-based recommendations for management. Eur J Endocrinol.

[84] Tsourdi E, Anastasilakis AD (2021). Parathyroid disease in pregnancy and lactation: a narrative review of the literature. Biomedicines.

[85] Hirsch PF (1967). Thyrocalcitonin inhibition of bone resorption induced by parathyroid extract in thyroparathyroidectomized rats. Endocrinology.

[86] Lewis P, Rafferty B, Shelley M, Robinson CJ (1971). A suggested physiological role of calcitonin: the protection of the skeleton during pregnancy and lactation. J Endocrinol.

[87] Taylor TG, Lewis PE, Balderstone O (1975). Role of calcitonin in protecting the skeleton during pregnancy and lactation. J Endocrinol.

[88] Žofková I (2016). Hypercalcemia. Pathophysiological aspects. Physiol Res.

[89] Laporta J, Keil KP, Weaver SR (2014). Serotonin regulates calcium homeostasis in lactation by epigenetic activation of hedgehog signaling. Mol Endocrinol.

[90] Kovacs CS (2021). Calcium and phosphate metabolism and related disorders during pregnancy and lactation. Endotext.

